# Shift in subsistence crop dominance from broomcorn millet to foxtail millet around 5500 BP in the western Loess Plateau

**DOI:** 10.3389/fpls.2022.939340

**Published:** 2022-07-26

**Authors:** Yishi Yang, Jia Wang, Gang Li, Jiajia Dong, Huihui Cao, Minmin Ma, Guoke Chen, Guanghui Dong

**Affiliations:** ^1^Gansu Provincial Institute of Cultural Relics and Archaeology, Lanzhou, China; ^2^MOE Key Laboratory of Western China’s Environmental System, College of Earth & Environmental Sciences, Lanzhou University, Lanzhou, China

**Keywords:** archaeobotanical analysis, radiocarbon dating, Gedachuan site, millet, late Neolithic, subsistence strategy.

## Abstract

Broomcorn and foxtail millet were the most important crops in northern China during the Neolithic period. Although the significance of broomcorn millet in human subsistence exceeded that of foxtail millet during the early Neolithic, this pattern was reversed by the end of Neolithic period. However, the process underlying this shift remains unclear. The recent excavation of the Gedachuan (GDC) in Zhangjiachuan county has revealed an abundance of relics including millet crop remains from relatively continuous strata of the Yangshao and Qijia cultures, and therefore provides a unique opportunity to examine how and when foxtail millet replaced broomcorn millet as the dominant crop in the western Loess Plateau during the Neolithic period. In this study, we identify 1,738 and 2,686 broomcorn and foxtail millet remains, respectively, from 74 flotation samples, accounting for 38.81% and 59.98% of total plant remains, respectively. Compared with 23 direct dates of carbonized crop grains in GDC, we propose that the weight of foxtail millet in plant subsistence of GDC first exceeded that of broomcorn millet as early as ∼5,500 BP, filling an important gap in the archaeobotanical record from the western Loess Plateau. Further comparative analysis of multidisciplinary data suggests the shift in significance of these two millet crops during the late Neolithic may have been triggered by variations in human settlement intensity and climate change in the western Loess Plateau. The results of this study also suggest that the Banpo Phase of Yangshao Culture survived in the western Loess Plateau as late as ∼5,600 BP.

## Introduction

Broomcorn and foxtail millet are among the oldest domesticated cereals in the world, first used in northern China around 10,000 BP (Zhao, 2011; Yang et al., 2012; Zhao et al., 2020). The cultivation of these two crops has been identified as one of the most important economic bases for the development of Neolithic cultures and emergence of ancient civilizations in northern China (e.g., Pearson and Underhill, 1987; Cao, 2006; Zhao, 2014; Han, 2021). However, recent evidence now suggests that the relative significance of broomcorn and foxtail millet in human subsistence changed throughout the Neolithic Age. Though broomcorn and foxtail millet were utilized in northern China during the pre-Yangshao period (∼10,000-6,000 BP) (e.g., Liu et al., 2012; Wang et al., 2017; Deng et al., 2020), hunting-gathering may have been the primary livelihood strategy in most areas during this period (Zhao, 2014; Dong et al., 2016a; Dong et al., 2022). Cultivation of millet crops became the most important subsistence strategy in the Loess Plateau during 7,000-6,000 BP (Zhao, 2014; Chen et al., 2016), and was widely expanded across the Yellow River valley during the late Neolithic period (Chen et al., 2015; Yang et al., 2021). Archaeobotanical evidence suggests that the significance of foxtail millet in plant subsistence was lower than broomcorn millet before ∼6,000 BP (Zhao, 2004; Wu et al., 2015; Dong et al., 2022), but became the dominant cultivated crop in northern China after 6,000 BP (Lee et al., 2007; Bao et al., 2018; Song et al., 2019; Yang et al., 2021; Dong et al., 2022). However, the precise timing and the context in which such a shift occurred are not yet fully understood due to the limited direct dates of millet remains between 7,000–5,000 BP.

The western Loess Plateau (WLP) has been identified as a key area for the development of rain-fed agriculture during the Neolithic Age (Cai, 2021). Remains of broomcorn millet have been recovered from the cultural layer of the Dadiwan Phase I (∼7,800-7,200 BP) (Liu et al., 2004; Li, 2018b), indicating that humans began utilizing millet crops in this area during the pre-Yangshao period. However, isotopic evidence indicates that millet crops did not contribute to human diets substantially before c. 5,900 BP, but had become the major staple after this time (Barton et al., 2009). Much archaeobotanical data in the WLP have been published in recent decades, mostly from the late Neolithic and Bronze Ages (e.g., Li et al., 2007; Jia et al., 2013; Chen et al., 2019; Chen et al., 2020). Current archaeobotanical evidence suggests foxtail and broomcorn millet were the primary and secondary crops, respectively, in the WLP between 5,000–4,000 BP. This differs from the earlier Dadiwan period (∼7,800-7,200 BP) when millet played a minor role in diets. The crop shift of millet farming probably occurred in the Miaodigou period of the Yangshao Culture (5,900-5,500 BP), largely based on archeological stratigraphy and typology (Qin, 2012). However, the chronological framework of the Yangshao Cultural system in the WLP is poorly understood due to the “old carbon effect” of radiocarbon dating for charcoals (Gavin, 2001; Yan, 2009; Dong et al., 2014). Therefore, direct radiocarbon dates of broomcorn millet and foxtail millet are lacking. The timing of the shift in crop dominance in the Neolithic WLP is enigmatic, largely due to the absence of archaeobotanical data from sites dated in 6,000-5,000 BP.

Nearly continuous stratum spanning the early, mid and late phases of Yangshao Culture (7,000-5,000 BP) and Qijia Culture (4,300-3,500 BP) have been uncovered from the excavation of the GDC site, providing a rare opportunity to explore the trajectory of plant subsistence in the WLP during the mid-late Neolithic period. Using identified plant remains, radiocarbon dating of crop remains unearthed from the GDC site, and their comparison to published data in the WLP, we aim to study the history of the transformation in cropping patterns, especially the shift in crop dominance from broomcorn millet to foxtail millet during the Yangshao and Qijia periods. Moreover, the chronology of the Banpo Phase in the WLP is vague due to the limited radiocarbon dates and “old carbon effect” of radiocarbon dating for charcoals. The GDC site is currently the largest Banpo Phase site in the WLP, and therefore, we also examined the chronology of the Banpo Phase of Yangshao Culture in this work.

## Study area

**FIGURE 1:**
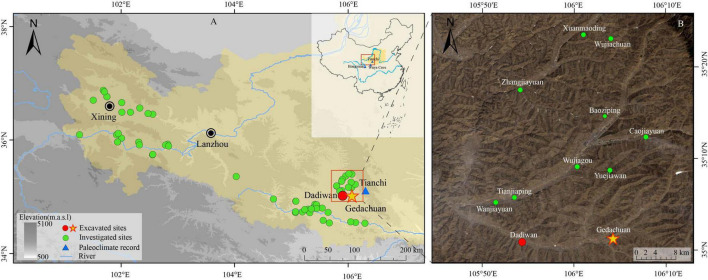
The distribution of survey and excavation sites in the WLP from 8,000 BP to 4,000 BP. **(A)** The distribution of survey and excavation sites in the WLP. **(B)** The distribution of Gedachuan site and its adjacent Zhuanglang county survey sites.

The western Loess Plateau (WLP) is situated to the west of Liupan Mountain, East of Wushaoling, North of Qinling, and is mainly situated in the middle of Gansu province (Figures 1A,B). The main rivers flowing through this area include the upper reaches of the Yellow River and the Wei River. The WLP is characterized by undulating ridges and gullies, with an arid climate, sparse vegetation, serious soil erosion and harsh ecological conditions. However, this area is associated with the development of several Neolithic cultures, including the Daiwan Phase I culture, and the Yangshao and Qijia cultures (The institute of archaeology CASS, 1999; Gansu Provincial Institute of Cultural Relics and Archaeology, 2006; Hosner et al., 2016). The GDC site is another important Yangshao Culture site in Zhangjiachuan county, which is situated at the intersection of the Nan and Songshu rivers, both of which are third-order tributaries of the Wei River. The site is surrounded by typical loess beam and loess gully landforms of the WLP. The climate of Zhangjiachuan county is classified as continental winter dry (Chan et al., 2016) and the mean annual temperature is 8-10°C with mean annual precipitation of 500-650 mm.

**FIGURE 2:**
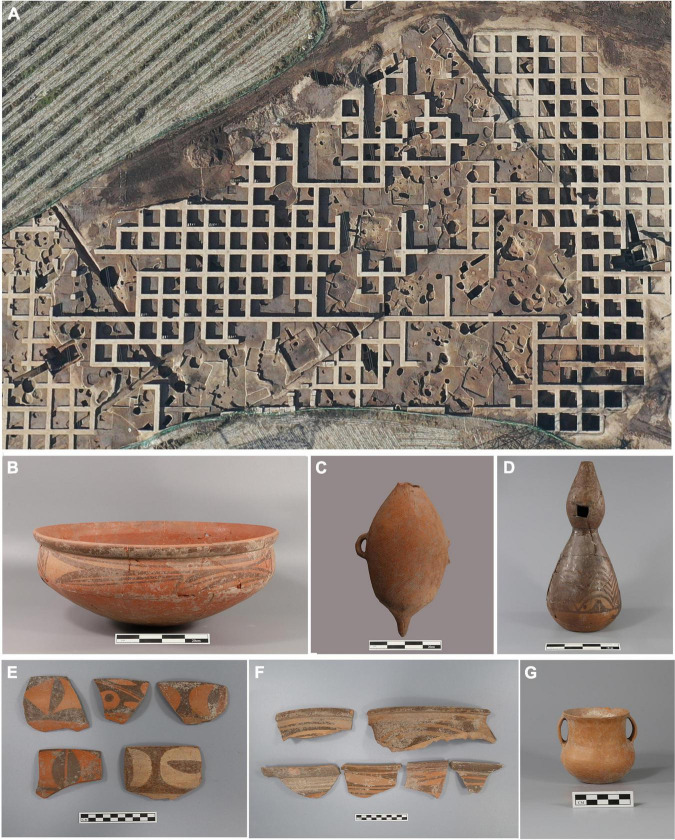
Excavation site of ring trench settlement and representative pottery of various cultural periods. **(A)** ring trench settlement. **(B–D)** pottery of the Banpo Phase of Yangshao Culture. **(E)** Painted pottery of the Maodigou Phase of Yangshao Culture. **(F)** Painted pottery of the Late Yangshao Culture. **(G)** Pottery of the Qijia Culture.

The GDC site was recently excavated in 2021, with an excavation area of about 27,000 m^2^,which preserved many rich cultural relics, including a complete Yangshao settlement. Neolithic relics include ash pits, houses, burials, kitchen pits, ditches, and kilns, and the relationship between the archeological strata is complex. According to the most recent excavations, the Neolithic settlement site stratums can be roughly divided into four periods: Banpo Phase of early Yangshao Culture, Miaodigou Phase of middle Yangshao Culture, late Yangshao Culture and Qijia Culture. Recently, the larger and well-preserved ring trench settlement in the Banpo Phase of Yangshao Culture was found (Figure 2A). This settlement included a residential area, burial area, and pottery kiln area, which is the same as the settlement of the Jiangzhai site (Peterson and Shelach, 2012). The unearthed artifacts classified as from the Banpo Phase mainly included pointy-bottom bottles, round bottom basins, round bottom bowls, tiny mouth tube belly tanks, gourd bottles, urn and jar covers (Figures 2B–D). In the middle-Yangshao Culture layer, remains from the Miaodigou Phase were damaged, of which a few individual houses and many ash pits were preserved and intact. The unearthed artifacts mainly included pointed bottom bottles, curved abdomen basins, bottom bowls, flat bottom bottles, urns and so on. Figure 2E shows the painted pottery basin of the Miaodigou Phase. The upper culture layer containing remains of the late Yangshao Culture and the Qijia Culture were mixed and seriously damaged by late human activities, of which only the house ground and some ash pits, kitchen pits, and ditches were preserved (Figures 2F,G).

## Material and methods

With the excavation of the GDC site, soil flotation samples were collected from cultural layers and relics units, such as houses, ash pits, kitchen pits, and ditches. Particular attention was paid to avoid disturbed contexts, specifically, samples were collected evenly for larger relic units like houses, which were sampled from different areas. Moreover, the volume of most soil samples was larger than 15 L, while the sampled volume of a few soil samples ranged from 3 L to 14 L (Supplementary Table 1). The collected soil samples were floated in a water-wave floatation machine. Here, the lighter remains, such as charcoal and charred plant seeds, floated upward and were gathered by an 80-mesh sifter (with a 0.2-mm aperture). The gathered material was subsequently wrapped in gauze and hung in a shady and cool area for desiccation. Next, the dried samples were sifted through 0.35, 0.7, 1.2, and 4 mm mesh sieves (Zhao, 2010). Finally, charred plant seeds were picked and identified in the Environment Archaeology Laboratory, Lanzhou University.

Carbonized crop seeds from the GDC site were pretreated with acid-base-acid washing processes and then graphitized using Auto Graphitization Equipment (AGE III), and finally were tested using a compact Accelerator Mass Spectrometer, Mini Carbon Dating System (MICADAS). These experiments were completed in the Radiocarbon Chronology Laboratory of Lanzhou University. Bayesian modeling of the ^14^C chronological data was performed using the built-in “Phase” function of the OxCal online program^1^ and IntCal20 curves (Reimer et al., 2020), using the “R_Date” function to enter the ^14^C dates. Each “Phase” included all ^14^C dates of a prehistoric culture, and each “Phase” function was bounded by the “Boundary” function. The start and end time of each prehistoric culture was constrained using this function, and the “Order” function was used to order the beginning and end of each prehistoric culture. Furthermore, the “outlier” function was used for each ^14^C chronological measurement (Long et al., 2017), to ensure that the model was reliable (Ramsey, 2009). The Bayesian model results were reported as a range of 95.4% and 68.3% and the median-to-median range was used to determine the chronological range of different prehistoric cultures (Long and Taylor, 2015; Yang et al., 2019).

## Results

### Archaeobotanical results

**FIGURE 3:**
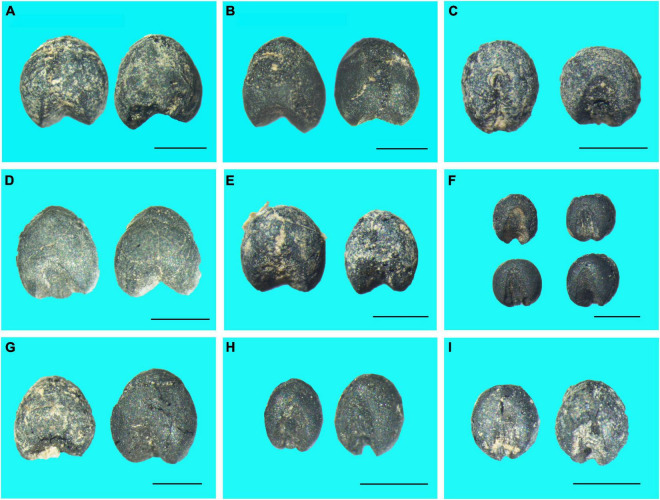
Photographs of charred crop remains unearthed from different periods at the Gedachuan site. **(A–C)** Banpo Phase. **(D–F)** Miaodigou Phase. **(G–I)** Qijia Culture. **(A,B,D,E,G)** Broomcorn millet. **(C,F,H,I)** Foxtail millet. (Scalebar: 1 mm).

The plant remains unearthed from the GDC site are listed in Supplementary Table 1, which includes 74 flotation samples from three periods, namely the Banpo Phase of early Yangshao Culture, the Miaodigou Phase of middle Yangshao Culture and the Qijia Culture. Overall, 1,475.5 L of flotation soil were floated, and a total of 4,428 charred crop seeds and 20 weed seeds were identified. As for crop seeds, the assemblage was composed of 2,686 foxtail millet (*Setaria italica*), 1,738 broomcorn millet (*Panicum miliaceum*), 2 rice (*Oryza sativa*) and 2 wheat (*Triticum aestivum*) seeds (Figure 3). A small number of weed seeds (20, representing 0.45% of the plant assemblage) were also recovered at the GDC site, including Oat (*Avena sativa*), Green foxtail (*Setaria viridis*), Milkvetch Root (*Astragalus adsurgens*), Daghestan Sweetclover (*Melilotus suaveolens*), Threehorned Badstraw (*Galium tricorne*), and Garden Sorrel (*Rumex acetosa*).

During the Banpo Phase, 138 foxtail millet seeds (13.28% of the plant assemblage), 895 broomcorn millet seeds (86.14% of the plant assemblage) and 2 wheat seeds (0.19% of the plant assemblage) were identified from 28 samples (574 L of soil in total). During the Miaodigou Phase, 115 foxtail millet seeds (23.23% of the plant assemblage) and 373 broomcorn millet seeds (75.35% of the plant assemblage) were identified from 32 samples (624.5 L of soil in total). Here, only one rice seed was identified. During the period of the Qijia Culture, 2,433 foxtail millet seeds (83.49% of the plant assemblage) and 470 broomcorn millet seeds (16.13% of the plant assemblage) were identified from 14 samples (277 L of soil in total). As in the Miaodigou period, only one rice seed was identified during the period of the Qijia Culture. The ratio of the number of identified foxtail and broomcorn millet seeds increased from 0.15 (Banpo Phase) and 0.31 (Miaodigou Phase) to 5.18 (Qijia Culture), indicating an increasing trend of foxtail millet cultivation importance in subsistence agriculture over time. In addition, a few weed seeds were identified in the Banpo, Miaodigou and Qijia Culture periods, accounting for 0.38%, 1.21% and 0.34% of the identified plant seeds, respectively.

### Radiocarbon dates

**FIGURE 4:**
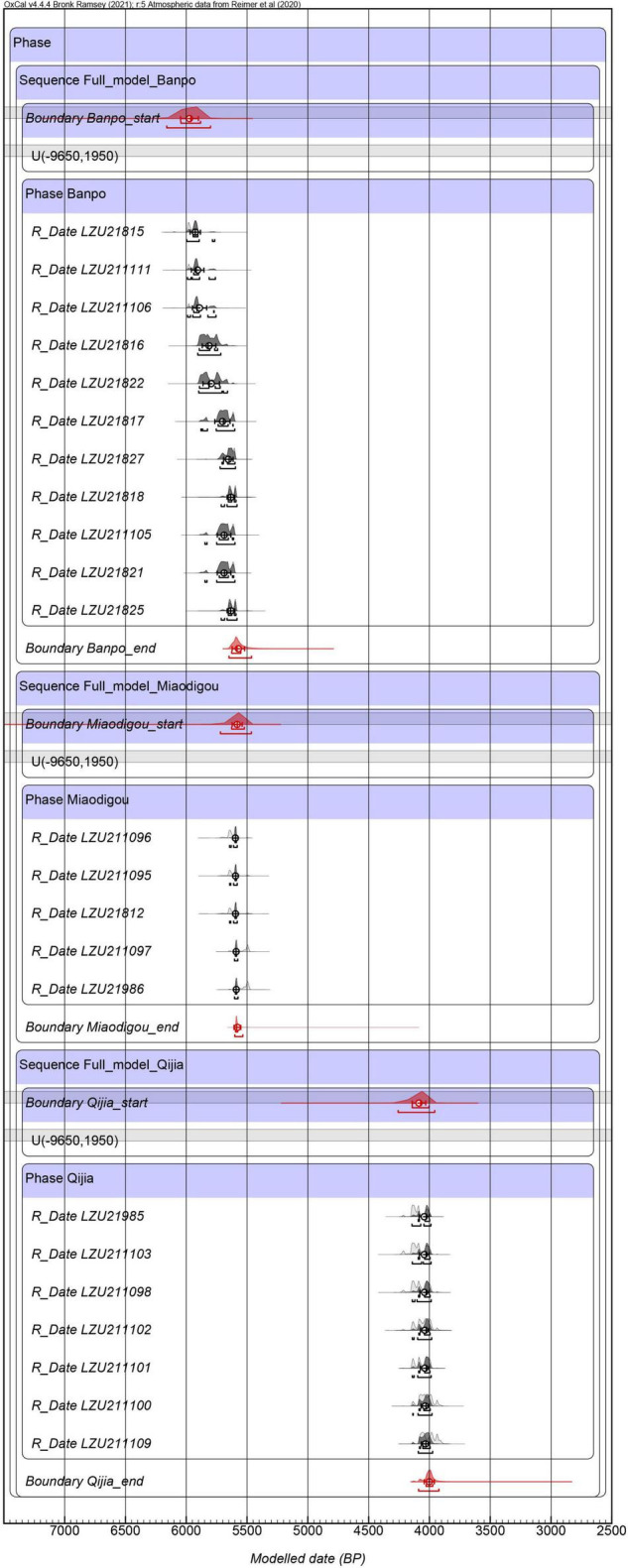
The chronology of different prehistoric cultures based on the Bayesian model at the Gedachuan site.

The 23 new ^14^C dates of charred seeds and charcoal with 95.4% age range are displayed in Table 1. According to the dating results, the site was divided into three periods. During the Banpo Phase, the dates of ten broomcorn millet seeds and one charcoal sample ranged from 5,997 to 5,581 cal yr BP. In the Miaodigou Phase, the dates of four broomcorn millet seeds and one foxtail millet seed ranged from 5,712 to 5,477 cal yr BP. In the period of the Qijia Culture, the dates of six foxtail millet seeds and one broomcorn millet seed ranged from 4,226 to 3,900 cal yr BP. The results from the Bayesian model are given for the Banpo Phase (5,970 BP∼5,570 BP), Miaodigou Phase (5,582 BP∼5,579 BP) and Qijia Culture (4,090 BP∼4,000 BP) (Figure 4).

**TABLE 1:** Calibrated radiocarbon dates of charred crop grains from the excavation of the Gedachuan site.

Lab Nu.	Sampling feature	Dating material	Dating method	Radiocarbon	Calibrated age (cal.yr BP)		Culture
				
				Age (yr BP)	1σ	2σ	
LZU21815	2021ZG IVT0502F15d5	Broomcorn millet	AMS	5,180 ± 30	5,988-5,911	5,997-5,900	Banpo
LZU211111	2021ZG IVT0703➂	Charcoal	AMS	5,160 ± 30	5,985-5,903	5,994-5,765	Banpo
LZU211106	2021ZGIT0203➃	Broomcorn millet	AMS	5,150 ± 30	5,985-5,897	5,992-5,758	Banpo
LZU21816	2021ZG II T0208F27z	Broomcorn millet	AMS	5,050 ± 30	5,893-5,742	5,904-5,719	Banpo
LZU21822	2021ZGIT0302F38d1	Broomcorn millet	AMS	5,030 ± 30	5,891-5,722	5,898-5,661	Banpo
LZU21817	2021ZG II T0207F25z	Broomcorn millet	AMS	4,980 ± 30	5,736-5,610	5,860-5,601	Banpo
LZU211105	2021ZG I T0203➂	Broomcorn millet	AMS	4,970 ± 30	5,725-5,609	5,844-5,600	Banpo
LZU21827	2021ZG II T1105F65d9	Broomcorn millet	AMS	4,930 ± 30	5,705-5,597	5,720-5,594	Banpo
LZU21818	2021ZG II T0207F25d3	Broomcorn millet	AMS	4,890 ± 30	5,652-5,589	5,712-5,581	Banpo
LZU21825	2021ZG I T0202F43d10	Broomcorn millet	AMS	4,890 ± 30	5,652-5,589	5,712-5,581	Banpo
LZU21821	2021ZGIT0202F38	Broomcorn millet	AMS	4,970 ± 30	5,725-5,609	5,844-5,600	Banpo
LZU211096	2021ZG II T0606H279	Broomcorn millet	AMS	4,890 ± 30	5,652-5,589	5,712-5,581	Miaodigou
LZU211095	2021ZG I T0101H276	Broomcorn millet	AMS	4,880 ± 30	5,652-5,586	5,705-5,488	Miaodigou
LZU21812	2021ZG II T1009F29	Broomcorn millet	AMS	4,880 ± 30	5,652-5,586	5,705-5,488	Miaodigou
LZU211097	2021ZG II T0610H275	Foxtail millet	AMS	4,840 ± 30	5,598-5,486	5,650-5,477	Miaodigou
LZU21986	2021ZG II T1206H64	Broomcorn millet	AMS	4,830 ± 30	5,596-5,483	5,600-5,477	Miaodigou
LZU21985	2021ZG II T0507H33	Broomcorn millet	AMS	3,740 ± 20	4,148-4,009	4,219-3,988	Qijia
LZU211103	2021ZG II T1704J20➃	Foxtail millet	AMS	3,740 ± 30	4,149-4,000	4,226-3,984	Qijia
LZU211098	2021ZG II T1009H190	Foxtail millet	AMS	3,730 ± 30	4,148-3,991	4,221-3,981	Qijia
LZU211102	2021ZG II T1409J15➃	Foxtail millet	AMS	3,710 ± 30	4,140-3,986	4,150-3,933	Qijia
LZU211101	2021ZG IV T0204H42	Foxtail millet	AMS	3,720 ± 20	4,142-3,992	4,148-3,984	Qijia
LZU211100	2021ZG IV T0604H38➁	Foxtail millet	AMS	3,690 ± 30	4,085-3,981	4,146-3,924	Qijia
LZU211109	2021ZG IV T0702H87	Foxtail millet	AMS	3,670 ± 30	4,082-3,929	4,090-3,900	Qijia

## Discussion

**FIGURE 5:**
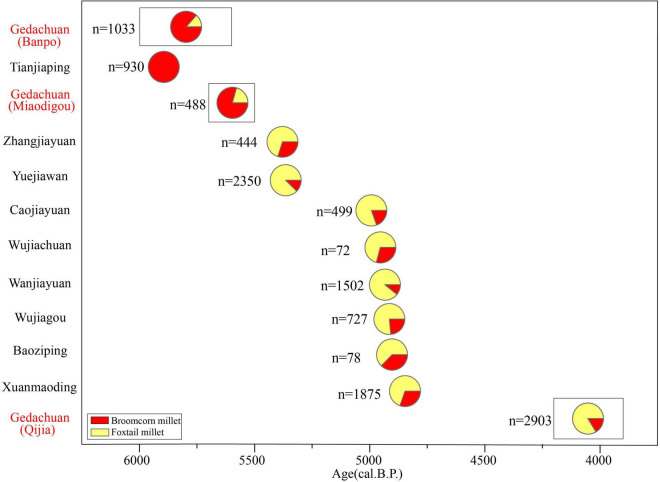
Proportion of crop remains from different periods at the Gedachuan site (in red) and the Zhuanglang survey sites (in black). Black font represents the survey sites with identified crop remains data from Li (2018). *n* represents the number of identified crop remains.

According to the archaeobotanical evidence identified from the GDC site (Figure 5 and Supplementary Table 1), the significance of foxtail millet as a subsistence crop gradually increased from the Banpo to the Qijia period, though broomcorn millet remained the most important cereal crop during the Banpo and Miaodigou phases. The proportional contribution of foxtail millet in a given assemblage evidently exceeded that of broomcorn millet following the late Yangshao period (∼5,500 BP) (Figure 5), which is consistent with previously published archaeobotanical data from other Neolithic sites in the WLP (e.g., Li, 2018b; Chen et al., 2019; Chen et al., 2020, Figures 6E,F).

**FIGURE 6:**
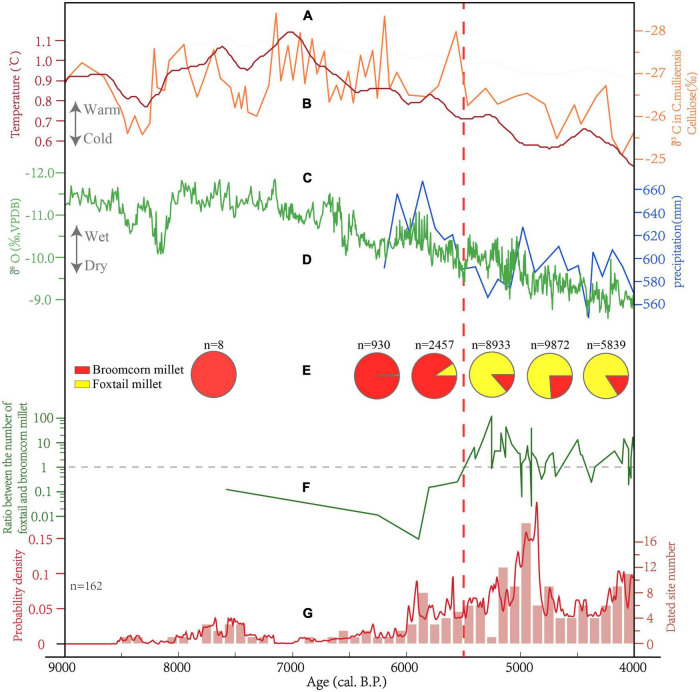
Comparison of human activity intensity, climate change and millet ratio in the WLP from 9,000–4,000 BP. **(A)** δ^13^C record of a single plant in Hongyuan peat bog (Hong et al., 2003). **(B)** Northern Hemisphere (30°∼90°N) temperature record (Marcott et al., 2013). **(C)** Reconstructed precipitation based on fossil pollen at Tianchi Lake (Zhao et al., 2010). **(D)** Stalagmite δ^18^O record at Wuya Cave (Tan et al., 2020). **(E)** Proportions of broomcorn millet and foxtail millet in the WLP between 9,000–4,000 BP at different periods. **(F)** Absolute number proportions of foxtail millet to broomcorn millet identified from sites dated between 9,000–4,000 BP in the WLP. **(G)** The Summed Probability Distribution (SPD) of averaged dates of bins (The line chart) and the dated site number (The column chart) in the WLP.

The utilization of broomcorn millet in the nearby Dadiwan site can be traced back to the 8^th^ millennium BP (Liu et al., 2004; Li, 2018b), while no evidence of foxtail millet during that period has been reported, which supports the superiority of broomcorn millet over foxtail millet during the pre-Yangshao period in the WLP. Similarly, this pattern has also been detected in the contemporaneous mid-lower reaches of the Yellow River, with the size of some broomcorn millets continuing to increase (Lu et al., 2009; Wu et al., 2015; Wang et al., 2017; Liu et al., 2018; Stevens et al., 2021), as well as in eastern Inner Mongolia (Zhao, 2004). Intensification of millet cultivation occurred in the Central Plains of northern China during the 7^th^ millennium BP (Zhao, 2014; Dong et al., 2016a; Dong et al., 2022), and the significance of foxtail millet in subsistence agriculture likely exceeded broomcorn millet in this area. However, the importance of broomcorn millet was still higher than foxtail millet in the WLP during the 7th millennium BP (Figures 6E,F), which is likely driven by the differences in the two taxa’s physiological responses to varying environments. Specifically, broomcorn millet is hardier than foxtail millet, in terms of drought-, frost- and barren tolerance (Editorial Department of Chinese Agricultural Encyclopedia, 1991; Li, 2018a; Dong et al., 2022), while foxtail millet is more high-yielding than broomcorn millet (Yang and Yu, 1992). The hydrothermal conditions of the WLP are much worse than those of Central Plains, which are more suitable to the cultivation of broomcorn millet in foothills close to rivers (Liu et al., 2009). Only a few sites in the WLP have been dated between 7,000-6,000 BP (Figure 6G), suggesting that human survival pressure was probably low in the area, considering the relatively low human settlement intensity and warm-wet climate during that period (Figures 6A–D).

The proportion of foxtail millet in cereal count overtook that of broomcorn millet in the GDC site after ∼5,500 BP based on the direct dates of the millet remains in the GDC site (Figure 5 and Table 1). This pattern contrasts previous archaeobotanical evidence from investigated sites in adjacent Zhuanglang county. During ∼6,000-5,400 BP, the abundance of broomcorn millet in flotation assemblages overshadowed foxtail millet at Tiannjiaping in Zhuanglang, for example. However, at other Zhuanglang county sites such as Zhangjiayuan and Yuejiawan, foxtail millet was more important. In 1 of 28 Banpo Phase (5,970 BP∼5,570 BP) samples and 1 of 32 Miaodigou Phase (5,582 BP∼5,579 BP) samples of the GDC site, the number of charred foxtail millet grains also exceeded those of broomcorn millet (Supplementary Table 1), while the total proportion of broomcorn millet remains far exceeded foxtail millet in these two phases (Figure 5). The disagreement in results of the archaeobotanical analysis at the GDC site and those of surveyed sites in Zhuanglang county dated between ∼6,000-5,400 BP are likely related to occasionality associated with small sample sizes in Zhuanglang county.

The percentage of foxtail millet in plant remains in the GDC site was clearly higher than broomcorn millet after ∼5,500 BP (Figure 5), indicating that foxtail millet was the most important subsistence crop in the WLP during the late Yangshao and Qijia periods. Similar trends have been previously observed in other WLP areas including Zhuanglang (Li, 2018b), Gangu (Chen et al., 2020), and Dingxi (Jia et al., 2013). Compared with the period of ∼6,000-5,500 BP, human settlement intensity in the WLP evidently increased during the period of ∼5,500-4,900 BP (Figure 6G), while temperature and precipitation obviously declined following ∼5,500 BP (Hong et al., 2003; Zhao et al., 2010; Marcott et al., 2013; Tan et al., 2020; Figures 6A–D). This may suggest that the adoption of higher-yielding foxtail millet over broomcorn in a context of a growing population and needing for agricultural intensification facing increasingly stressful environments (Liu and Reid, 2020). In general, foxtail millet yield is much higher than broomcorn millet yield. The average crop yields in China report that broomcorn millet yield is only 750-1,500 kg/ha, while foxtail millet yield is as high as 2,250-3,750 kg/ha (Chai and Feng, 2003). Especially in the Loess Plateau, foxtail millet yield is almost twice as high as broomcorn millet yield. At a broader geographic level, foxtail millet had become a dominant crop over taking broomcorn millet in other parts of north China after 6,000 BP (Lee et al., 2007; Bao et al., 2018; Song et al., 2019; Yang et al., 2021).

Besides changes in the environment and survival pressure, the assemblage formation process might influence cropping patterns. For instance, grain ubiquity could be driven by crop processing with variations in post-harvest labor or the scale of processing could influence crop percentages (e.g., Stevens, 2003; Van der Veen and Jones, 2006). However, as these effects were not directly investigated here, future work is required to determine the influence of crop processing on cropping patterns.

The 11 radiocarbon dates of millet remains from the Banpo cultural layers in the GDC site ranged from 5,997 to 5,581 BP (Table 1), which is a few hundred years later than previously estimated (Banpo: 7,000-5,900 BP) (Dai, 1998; Gansu Provincial Institute of Cultural Relics and Archaeology, 2006; Zhang et al., 2013). With the application of the Bayesian model, the chronology of the Banpo samples in the GDC site was refined to ∼5,970-5,570 BP. As typical Banpo style ceramics, featuring double-gourd vase, pointed bottom bottle and red pottery bowl have been unearthed from the associated stratigraphic units, we considered the cultural association with the Banpo Phase was reliable (Figures 2B–D). The new radiocarbon results reported here shed insights into the variation of the Banpo chronology in WLP, notwithstanding of the potential “old wood” effect resulting from charcoal dates. (Gavin, 2001; Yan, 2009; Dong et al., 2014). Similarly, the difference of chronologies of the Neolithic and Bronze Age estimated between short-lived crop remains, bones and other various materials has also been reported in the Hexi Corridor (Yang et al., 2019), Qaidam basin (Dong et al., 2016b) and Haidai region (Long et al., 2017).

## Conclusion

The new archaeobotanical and radiocarbon dating data presented in this study suggest that the shift in crop dominance from broomcorn millet to foxtail millet in the WLP occurred following ∼5,500 BP, with broomcorn millet becoming the primary subsistence crop during the period of ∼6,000-5,500 BP, and foxtail millet becoming the most important crop in the subsequent period of ∼5,500-4,000 BP. The relative importance of foxtail millet to broomcorn millet increased in a stepwise fashion in the WLP from the Banpo, MiaoDigou, late Yangshao phases into the period of Qijia Culture, which was likely triggered by the aggravation of human survival pressure in relation to human settlement intensity and climate change. Based on the analysis of new radiocarbon dates of millet remains from the Banpo cultural layers at the GDC site, we estimate the chronology of the Banpo Phase of Yangshao Culture in the WLP to ∼6,000-5,600 BP, although additional investigation is required in the future.

## Data availability statement

The original contributions presented in this study are included in the article/Supplementary material, further inquiries can be directed to the corresponding author/s.

## Author contributions

GD, GC, and MM designed the study. YY, JW, GL, and JD conducted the field surveys and sample collection. YY, JW, HC, GL, and JD completed the experiments and data correction. YY, JW, HC, and MM analyzed the data and designed the figures. YY, JW, and MM wrote the manuscript in consultation with all authors. All authors discussed the results and commented on the manuscript.

## Footnotes

1
https://c14.arch.ox.ac.uk/oxcal.html
