# Supplementary material for: Shift in subsistence crop dominance from broomcorn millet to foxtail millet around 5500 BP in the western Loess Plateau
Source: Front Plant Sci. 2022 Jul 26;13:939340. doi: 10.3389/fpls.2022.939340 (PMC9362993; doi:10.3389/fpls.2022.939340)
Supplement: Supplementary file 1 [file Table_1.docx]

**Supplementary Table 1.** Seed Identification Results of Plants at the Gedachuan site

| **Culture** | **Sample number** | **Flotation volume/L** | **Setaria italica** | **Panicum miliaceum** | **Oryza sativa** | **Triticum aestivum** | **Avena sativa** | **Setaria viridis** | **Astragalus adsurgens** | **Melilotus suaveolens** | **Galium tricorne** | **Rumex acetosa** | **Unknown weed** | **Total** |
| --- | --- | --- | --- | --- | --- | --- | --- | --- | --- | --- | --- | --- | --- | --- |
| **Banpo** | 2021ZG IVT0502F15d5 | 5 |  | 8 |  |  |  |  |  |  |  |  |  | **8** |
|  | 2021ZG IVT0502F15z | 15 | 2 | 3 |  |  |  |  |  |  |  |  |  | **5** |
|  | 2021ZGⅡT0207F25z | 15 | 2 | 76 |  |  |  |  |  |  |  |  |  | **78** |
|  | 2021ZGⅡT0207F25d3 | 22 |  | 9 |  |  |  |  |  |  |  |  |  | **9** |
|  | 2021ZGⅡT0208F27Z1 | 12 | 1 | 6 |  |  |  |  |  |  |  |  |  | **7** |
|  | 2021ZGⅡT0208F27 | 25 |  | 28 |  |  |  |  |  |  |  |  |  | **28** |
|  | 2021ZGⅡT0208F27d1 | 15 | 2 | 12 |  |  |  |  |  |  |  |  |  | **14** |
|  | 2021ZGⅡT0609F37 | 21 | 5 | 33 |  |  |  |  |  |  |  |  |  | **38** |
|  | 2021ZGⅡT0609F37d1 | 14 | 4 | 28 |  |  |  | 1 |  |  |  |  |  | **33** |
|  | 2021ZGIT0202F38 | 22 | 3 | 45 |  |  |  |  |  |  |  |  |  | **48** |
|  | 2021ZGIT0202F38d1 | 28 | 5 | 41 |  |  |  |  |  |  |  |  |  | **46** |
|  | 2021ZGⅠT0807F42z | 21 | 6 | 18 |  |  |  |  |  |  |  |  |  | **24** |
|  | 2021ZGⅠT0202F43z | 21 |  | 38 |  |  |  |  |  |  |  |  |  | **38** |
|  | 2021ZGⅠT0202F43d10 | 17 | 3 | 30 |  |  |  |  |  |  |  |  |  | **33** |
|  | 2021ZGⅡT1105F65 | 14 | 1 | 12 |  |  |  |  |  |  |  |  |  | **13** |
|  | 2021ZGⅡT1105F65d9 | 47 |  | 47 |  |  |  |  |  |  |  |  |  | **47** |
|  | 2021ZGⅠT1210G8 | 22 | 8 | 20 |  |  |  |  |  |  |  |  |  | **28** |
|  | 2021ZGⅠT1307G8 | 21 | 12 | 220 |  |  |  |  |  |  |  |  |  | **232** |
|  | 2021ZGⅠT1111G8 | 21 | 2 | 13 |  |  |  |  |  |  |  |  |  | **15** |
|  | 2021ZGⅣT1006G8 | 14 |  | 22 |  |  |  |  |  |  |  |  |  | **22** |
|  | 2021ZGⅠT1003H474 | 19 | 1 | 6 |  |  |  |  |  |  |  |  |  | **7** |
|  | 2021ZGⅠT0405H467 | 21 | 4 | 52 |  |  |  |  |  |  |  |  | 1 | **57** |
|  | 2021ZGⅠT0505H501 | 21 | 36 | 68 |  |  | 1 |  |  |  |  |  | 3 | **108** |
|  | 2021ZGⅠT0510H566 | 16 | 2 | 3 |  |  |  |  |  |  |  |  |  | **5** |
|  | 2021ZGⅡT1110H494 | 24 | 36 | 36 |  | 2 | 1 |  |  |  |  |  |  | **75** |
|  | 2021ZGⅡT1304H519 | 24 | 1 | 13 |  |  |  |  |  |  |  |  | 1 | **15** |
|  | 2021ZGⅡT1403H522 | 32 | 1 | 3 |  |  |  |  |  |  |  | 1 |  | **5** |
|  | 2021ZGⅡT0709H491 | 25 | 1 | 5 |  |  |  |  |  |  |  |  |  | **6** |
| **Miaodigou** | 2021ZGⅠT0301F9F9 | 6 |  | 2 |  |  |  |  |  |  |  |  |  | **2** |
|  | 2021ZGⅠT0502F10z③ | 3 |  | 2 |  |  |  |  |  |  |  |  |  | **2** |
|  | 2021ZGⅠT0502F22 | 32 | 2 | 4 |  |  |  |  |  |  |  |  |  | **6** |
|  | 2021ZGⅢT0101F23① | 21 |  | 5 |  |  |  |  |  |  |  |  |  | **5** |
|  | 2021ZGⅢT0101F23z | 22 |  | 3 |  |  |  |  |  |  |  |  |  | **3** |
|  | 2021ZGⅢT0101F23② | 22 |  | 5 |  |  |  |  |  |  |  |  |  | **5** |
|  | 2021ZGⅠT0204F28d1 | 15 | 1 | 8 |  |  |  |  |  |  |  |  |  | **9** |
|  | 2021ZGⅡT1009F29① | 15 | 3 | 15 |  |  |  |  |  |  |  |  |  | **18** |
|  | 2021ZGⅡT1009F29② | 14 | 18 | 32 |  |  |  |  |  |  |  |  |  | **50** |
|  | 2021ZGⅡT1009F29 | 22 | 5 | 22 |  |  |  |  |  |  |  |  |  | **27** |
|  | 2021ZGⅡT1009F29d1 | 15 | 9 | 12 |  |  |  |  |  |  |  |  |  | **21** |
|  | 2021ZGⅡT0405H202 | 18 |  | 8 |  |  |  |  |  |  |  |  |  | **8** |
|  | 2021ZGⅠT0204H248 | 24 |  | 5 |  |  |  |  |  |  |  |  |  | **5** |
|  | 2021ZⅡT0104H228 | 18 | 1 | 2 |  |  |  |  |  |  |  |  |  | **3** |
|  | 2021ZGⅠT0404H287① | 21 | 7 | 20 |  |  |  |  |  |  |  |  | 1 | **28** |
|  | 2021ZGⅠT0301H229 | 27 | 3 | 1 |  |  |  |  |  |  |  |  | 1 | **5** |
|  | 2021ZGⅡT1009H191 | 15 | 6 | 18 |  |  |  |  |  | 1 |  |  |  | **25** |
|  | 2021ZGⅠT0101H276 | 23 | 7 | 28 |  |  |  |  |  |  |  |  |  | **35** |
|  | 2021ZGⅡT0606H279 | 29 | 10 | 60 |  |  |  |  |  |  | 1 | 4 |  | **75** |
|  | 2021ZGⅡT0504H199 | 18 | 1 | 4 |  |  |  |  |  |  |  |  |  | **5** |
|  | 2021ZGⅡT0803H283 | 26 |  | 17 |  |  |  |  |  |  |  |  |  | **17** |
|  | 2021ZGT0705H168 | 21 | 1 | 2 |  |  |  |  |  |  |  |  | 1 | **4** |
|  | 2021ZGⅡT0504H173 | 25 | 2 | 3 |  |  |  |  |  |  |  |  |  | **5** |
|  | 2021ZGⅡT1206H64 | 8.5 | 1 | 9 |  |  |  |  |  |  |  |  | 6 | **16** |
|  | 2021ZGⅡT0404H175 | 21 | 2 | 3 | 1 |  |  |  |  |  |  |  | 1 | **7** |
|  | 2021ZGⅡT1009H192 | 25 | 3 | 28 |  |  |  |  |  |  |  |  |  | **31** |
|  | 2021ZGⅡT1207H219 | 17 | 4 | 8 |  |  |  |  |  |  |  |  |  | **12** |
|  | 2021ZGⅡT0408H273 | 23 | 6 | 16 |  |  |  |  |  |  |  |  | 1 | **23** |
|  | 2021ZGⅡT0803H251 | 23 |  | 3 |  |  |  |  |  |  |  |  |  | **3** |
|  | 2021ZGⅡT0406H213 | 17 |  | 4 |  |  |  |  |  |  |  |  |  | **4** |
|  | 2021ZGⅡT0810H303 | 14 |  | 4 |  |  |  |  |  |  |  |  |  | **4** |
|  | 2021ZGⅡT0610H275 | 24 | 23 | 20 |  |  |  |  |  |  |  |  | 2 | **45** |
| **Qijia** | 2021ZGⅡT1409J15① | 25 |  | 2 |  |  |  |  |  |  |  |  |  | **2** |
|  | 2021ZGⅡT1409J15② | 21 | 102 | 20 |  |  |  |  |  |  |  |  | 1 | **123** |
|  | 2021ZGⅡT1409J15③ | 21 | 47 | 13 |  |  |  |  | 2 |  |  |  | 1 | **63** |
|  | 2021ZGⅡT1409J15④ | 27 | 75 | 23 |  |  |  | 2 |  |  |  |  | 1 | **101** |
|  | 2021ZGⅡT1704J20① | 21 | 48 | 18 |  |  |  |  |  |  |  |  | 2 | **68** |
|  | 2021ZGⅡT1704J20② | 27 | 482 | 72 | 1 |  |  |  |  |  |  |  |  | **555** |
|  | 2021ZGⅡT1704J20③ | 21 | 102 | 22 |  |  |  |  |  |  |  |  |  | **124** |
|  | 2021ZGⅡT1704J20④ | 23 | 362 | 35 |  |  |  |  | 2 |  |  |  | 2 | **401** |
|  | 2021ZGⅡT1704J20⑤ | 12 | 138 | 20 |  |  |  |  |  |  |  |  |  | **158** |
|  | 2021ZGⅡT1704J20⑤ | 23 | 423 | 52 |  |  |  |  | 1 |  |  |  | 2 | **478** |
|  | 2021ZGⅡT0507H33 | 12 | 28 | 13 |  |  |  |  |  |  |  |  |  | **41** |
|  | 2021ZGⅡT1009H190 | 27 | 78 | 48 |  |  |  |  | 2 |  |  |  | 1 | **129** |
|  | 2021ZGⅣT0604H38① | 9 | 46 | 2 |  |  |  |  |  |  |  |  |  | **48** |
|  | 2021ZGⅣT0604H38② | 8 | 502 | 130 |  |  |  |  | 1 |  |  |  | 2 | **635** |
| **Total** |  | **1475.5** | **2686** | **1738** | **2** | **2** | **2** | **3** | **8** | **1** | **1** | **5** | **30** | **4478** |
